# Supplementary material for: A model of bi-directional interactions between complementary learning systems for memory consolidation of sequential experiences
Source: Front Syst Neurosci. 2022 Oct 13;16:972235. doi: 10.3389/fnsys.2022.972235 (PMC9606815; doi:10.3389/fnsys.2022.972235)
Supplement: Supplementary file 1 [file Data_Sheet_1.pdf]

Supplementary Material:

A model of bi-directional interactions between complementary learning systems for memory consolidation of sequential experiences

Michael D. Howard, Steven W. Skorheim, and Praveen K. Pilly*
*Correspondence:
Praveen K. Pilly
pkpilly@hrl.com

1 SUPPLEMENTARY FIGURES

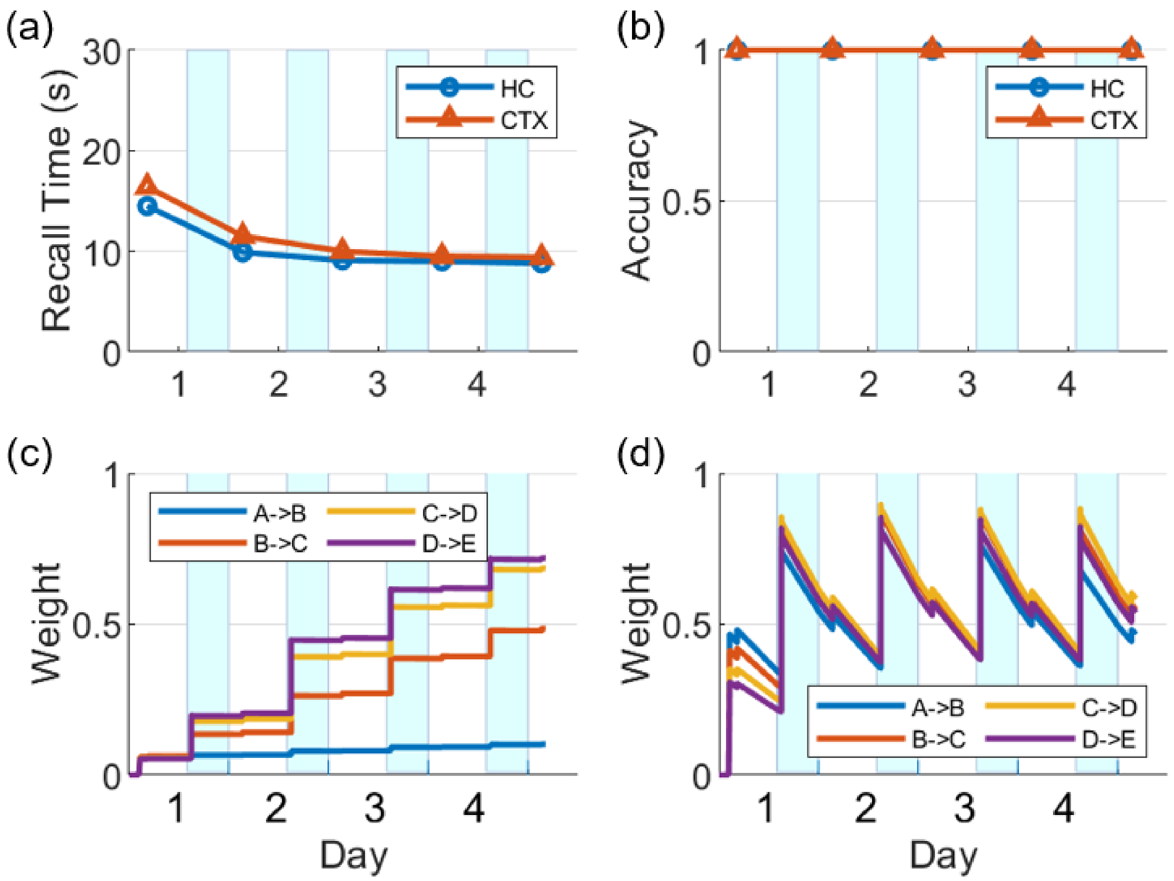

Figure S1. Higher recall threshold. Replication of the experiment shown in Figure 8 for the baseline network with the recall threshold raised to 0.05 (compared to the default value of 0.01). (a) Recall time, where 30 s is default for incomplete recalls. (b) Recall accuracy. (c) Weight strengths in the cortex. (d) Weight strengths in the hippocampus.

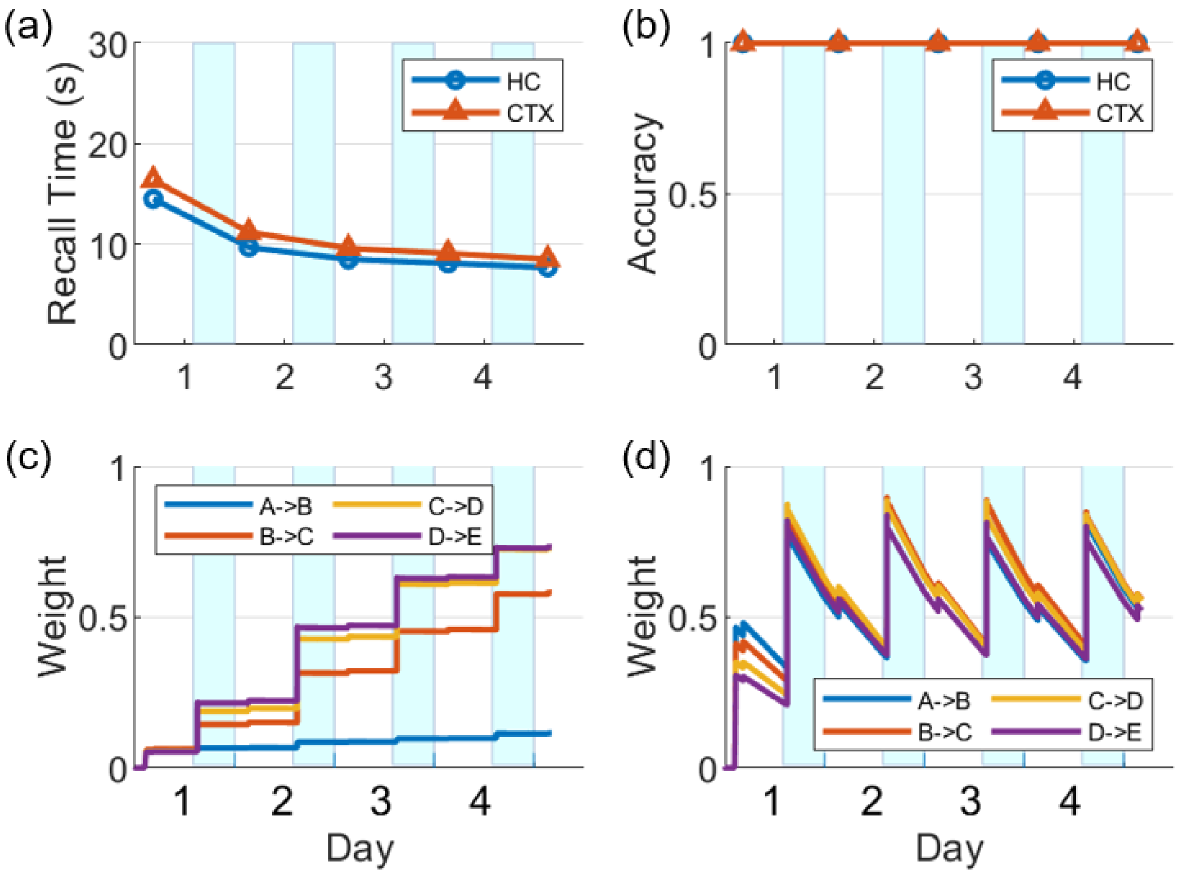

Figure S2. Random initial weights. Replication of the experiment shown in Figure 8 for the baseline network with all weights randomly initialized using a uniform distribution on the interval $[0, 0.05]$. (a) Recall time, where 30 s is default for incomplete recalls. (b) Recall accuracy. (c) Weight strengths in the cortex. (d) Weight strengths in the hippocampus.

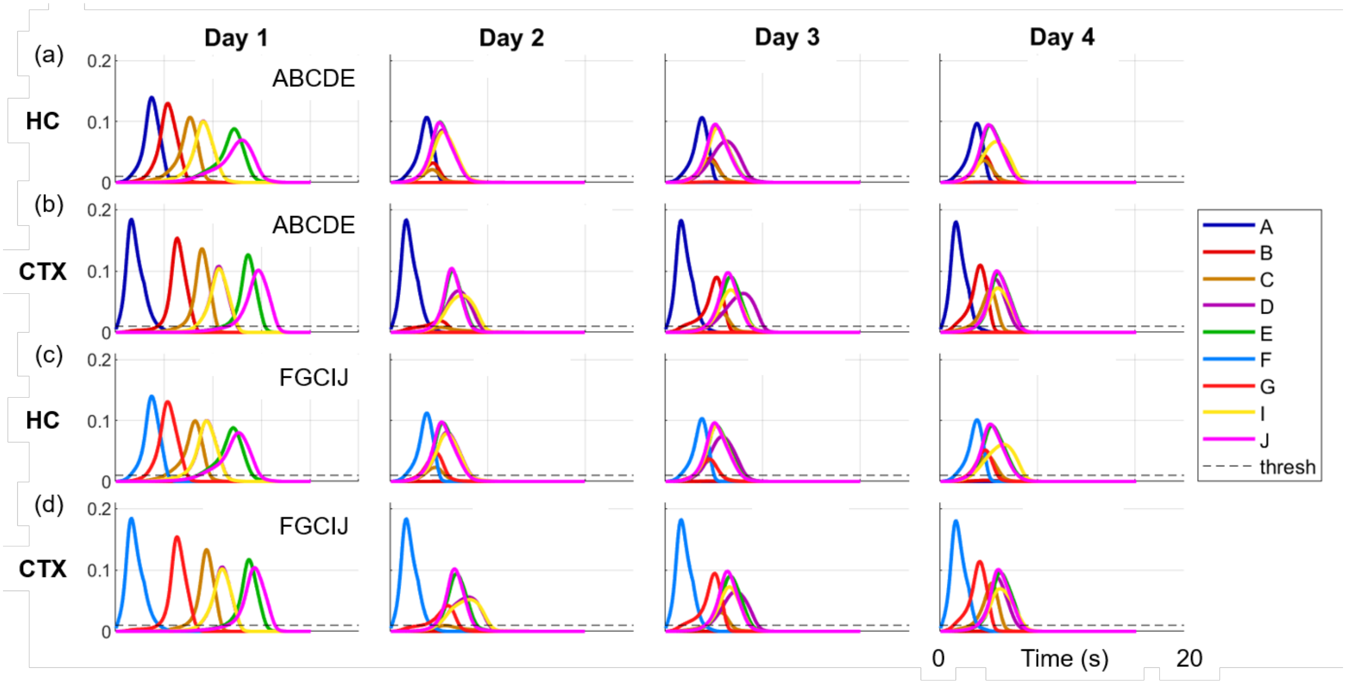

Figure S3. Waking recalls of two sequences that overlap in the third item during a four-day, four-night simulation. Sequences ABCDE and FGCIJ were trained for 10 trials each on four consecutive days (Days 1-4). 50 cycles of 1 Hz SWO were run each of the four nights (Nights 1-4). Memory recall tests were conducted at the end of each day. (a) ABCDE recall in the hippocampus (HC) each day. (b) ABCDE recall in the cortex (CTX) each day. (c) FGCIJ recall in HC each day. (d) FGCIJ recall in CTX each day.

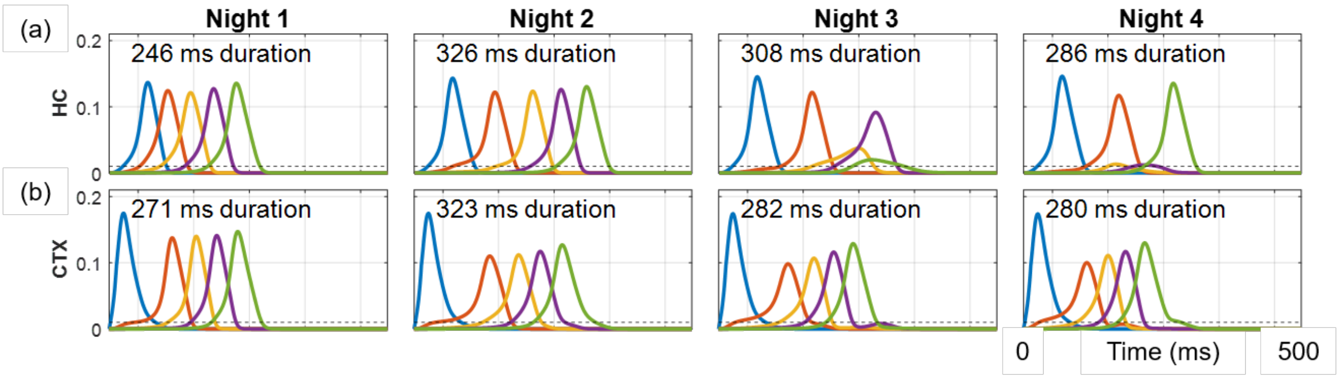

Figure S4. Representative replays for the simulation experiment where hippocampal learning is turned off during sleep (shown in Figure 11). The last full replay, which is cued by the first item, is plotted here for each of the four nights in the (a) hippocampus (HC) and (b) cortex (CTX) in the context of the 500 ms UP state. The duration of replay is printed at the top of each plot.
